# High-resolution T2-FLAIR and non-contrast CT brain atlas of the elderly

**DOI:** 10.1038/s41597-020-0379-9

**Published:** 2020-02-17

**Authors:** Deepthi Rajashekar, Matthias Wilms, M. Ethan MacDonald, Jan Ehrhardt, Pauline Mouches, Richard Frayne, Michael D. Hill, Nils D. Forkert

**Affiliations:** 1 Biomedical Engineering Graduate Program, University of Calgary, Calgary, AB Canada; 2 Department of Radiology, Cumming School of Medicine, University of Calgary, Calgary, AB Canada; 3 Healthy Brain Aging Lab, University of Calgary, Calgary, AB Canada; 4 Institute of Medical Informatics, University of Luebeck, Lübeck, Germany; 5 Seaman Family MR Research Center, Foothills Medical Centre, Calgary, AB Canada; 6 Calgary Image Processing and Analysis Center (CIPAC), Foothills Medical Centre, Calgary, AB Canada; 7 Department of Clinical Neurosciences, Cumming School of Medicine, University of Calgary, Calgary, AB Canada

**Keywords:** Medical research, Anatomy

## Abstract

Normative brain atlases are a standard tool for neuroscience research and are, for example, used for spatial normalization of image datasets prior to voxel-based analyses of brain morphology and function. Although many different atlases are publicly available, they are usually biased with respect to an imaging modality and the age distribution. Both effects are well known to negatively impact the accuracy and reliability of the spatial normalization process using non-linear image registration methods. An important and very active neuroscience area that lacks appropriate atlases is lesion-related research in elderly populations (e.g. stroke, multiple sclerosis) for which FLAIR MRI and non-contrast CT are often the clinical imaging modalities of choice. To overcome the lack of atlases for these tasks and modalities, this paper presents high-resolution, age-specific FLAIR and non-contrast CT atlases of the elderly generated using clinical images.

## Abstract (summary)

**Table:** 

Measurement(s)	neuroimaging measurement • Brain group average • Elderly
Technology Type(s)	Computed Tomography of the Brain without Contrast • Fluid Attenuated Inversion Recovery • Image Registration
Sample Characteristic - Organism	Homo sapiens

Machine-accessible metadata file describing the reported data: 10.6084/m9.figshare.11662959

## Background & Summary

Brain atlases are an indispensable tool for neuroscience research and clinical purposes. Brain atlases are commonly used for spatial normalization of image datasets acquired from different patients to remove individual shape variations prior to voxel-based analyses. They are also used for atlas-based analysis of brain morphology and function in patients using pre-defined regions of interest in the atlases.

Many atlases are biased with respect to the imaging modality used for atlas generation^1,2^. They are also limited regarding the age distribution and health status of the subjects used for atlas generation. Both, the modality and age distribution, can pose considerable problems for neuroimaging studies and clinical applications that require accurate spatial correspondences between the patient scans and the atlas (typically determined using non-linear image registration methods). Previous studies have shown that the registration accuracy has a significant effect on the results of neuroimaging studies using traditional image analysis methods as well as state-of-the-art deep learning approaches^3–6^. Within this context, substantial shape differences between the images (*e.g*., atrophy caused by brain age or neurological diseases^7^) and image appearance differences (*i.e*., contrast differences across imaging modalities) are known to have a considerable impact on the accuracy of brain image registration. Determining the large deformations required in case of considerable atrophy differences between the atlas subjects and patient scans is not trivial. The registration of images obtained using different modalities can be sub-optimal due to appearance differences of corresponding brain structures that are not appropriately captured by the similarity metric.

For neurological studies, the Montreal Neurologic Institute (MNI) reference atlas is the most common reference space definition used. In this space, datasets from a healthy young adult cohort with an average age of 23 years were iteratively and non-linearly transformed to a common space. These atlases are available for T1-, T2-, and proton density (PD) weighted magnetic resonance imaging (MRI)^8,9^. While very useful, these atlases are not ideal when analyzing datasets acquired from elderly cohorts, which is often the case when investigating neurological diseases. To overcome this limitation, various atlases using datasets of elderly subjects have been generated and made available. A popular example is the groupe d’imagerie neurofonctionnelle (GIN) atlas^10^, a probabilistic T1-weighted MRI template of subjects between 63 and 75 years, displaying gray matter and white matter atrophy, and increased cerebrospinal fluid (CSF) volume.

Atlases derived from standard T1-, T2-, or PD-weighted MRI datasets are usually suboptimal for atlas-based analysis or registration of other image modalities. Within this context, fluid-attenuated inversion recovery (FLAIR) MR and non-contrast computed tomography (NCCT) datasets are two common clinically used image modalities and, therefore, especially important for neuroscience research and development of computer-aided diagnosis tools. Common applications of FLAIR and NCCT datasets include lesion segmentation (*e.g*. stroke, multiple sclerosis) that can be used for lesion-symptom mapping^11^, while non-contrast CT datasets are also typically used for determining the Alberta stroke programme early CT (ASPECT) ^12^, for surgical planning of image-guided deep brain stimulation of various neurological diseases, as well as lesion-symptom mapping^11^. Corresponding T1- or T2-weighted MRI datasets that could be used for intermediate image processing steps are often not acquired in these patients. Furthermore, registration of the low-resolution pathology-specific image modalities to the T1-weighted MR scan, which is then registered to the age-biased atlases, often leads to poor results using the standard brain atlases.

The number of available elderly FLAIR and NCCT brain atlases is limited. As the only prominent example, the widely used Statistical Parametric Mapping (SPM) clinical toolbox (https://www.nitrc.org/projects/clinicaltbx/) includes a T1-weighted MRI and contrast-enhanced CT (CECT) atlas, constructed using datasets of subjects with average ages of 72.9 years and 61.3 years, respectively. The SPM clinical toolbox also contains a low-resolution FLAIR and CECT atlas in the same image coordinate space. The high-resolution version of this FLAIR atlas was generated using datasets of a much larger cohort with ages ranging from 18–85 years, originally employed for generation of the Brainder atlas^13^. The CECT atlas contains high-intensity vessel information that is likely to impact registration accuracy of structural quantitative NCCT scans. Despite the advances in non-linear symmetric registration techniques used for atlas generation, both atlases (FLAIR and CECT) are comparably blurry. The lack of high-frequency edge information across brain tissues and brain regions represents a considerable problem for accurate spatial normalisation of individual patient datasets.

**Fig. 1:**
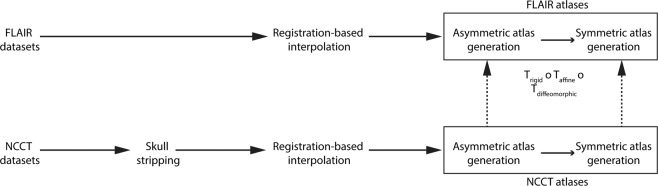
Processing pipeline for generating high-resolution FLAIR and NCCT atlases.

Therefore, the main objective of this work was to generate high-resolution brain atlases using standard low-resolution, clinical image datasets of elderly subjects acquired with clinically relevant image modalities. The proposed atlases are referred to in the following as the Medical Image Analysis and Machine Learning Laboratory (MIPLAB) FLAIR and non-contrast computed tomography (NCCT) brain atlases. The pipeline for creating these age-specific FLAIR and NCCT atlases for the elderly is shown in Fig. 1. Furthermore, the secondary objective of this work was to provide symmetric versions of the atlases (following the standard set by ICBM 152 nonlinear atlases^14^), which are devoid of any inherent asymmetry of the brain resulting from hemispheric differences due to tissue and the scanner biases.

## Methods

### Datasets

Two databases were used to generate the atlases in this work. The FLAIR MRI scans were obtained from the Calgary Normative Study^15,16^. A total of 136 healthy elderly subjects (no neurological disease) with an average age of 66 ± 13 years (mean ± standard deviation) were used for FLAIR atlas generation. All FLAIR MRI scans were acquired using a clinical 3 T scanner (Discovery 750w, GE Healthcare) employing an inversion recovery T2-weighted sequence with TI/TR/TE/flip angle of 2250 ms/9000 ms/145 ms/90°, a 256 × 256 matrix size, in-plane resolution of 0.94 mm, and 3.0 mm slice thickness.

For NCCT atlas generation, 47 admission NCCT scans of patients with average age of 72 ± 14 years with acute ischemic stroke but without any visible lesions or early ischemic change corresponding to an ASPECT score of 10 were obtained from the ESCAPE trial^17^. The CT scans used for NCCT atlas generation were obtained from multiple scanners at various sites with slice thickness varying between 3–5 mm and the in-plane resolution varying between 0.38 mm – 0.5 mm acquired using 120 kEV tube potential. None of the patients included received contrast administration prior to CT image acquisition.

Acquisition of the datasets for the two trials was approved by the respective local ethics board at each site contributing to the two trials. All datasets used in this retrospective and secondary study were made available after complete anonymization so that additional ethics approval and/or informed consent was not required.

### Pre-processing

The main objective of the pre-processing of the datasets was to generate isotropic 1 mm^3^ high-resolution datasets of the individual low-resolution images. Since standard (linear) interpolation would introduce severe topological artifacts (*e.g*. holes, new structures, etc.) due to the large inter-slice distances in superior-inferior direction of the clinical NCCT and FLAIR datasets, a specialized registration-based interpolation method was used^18^. The main idea of this approach is to first compute spatial correspondences between adjacent axial slices via diffeomorphic image registration^19^ and then to perform (linear) interpolation along the estimated displacement vectors. By doing so, the risk of mixing intensity values from different tissue classes is reduced. Thus, the topology of the structures is preserved and much sharper images can be obtained in comparison to the standard interpolation techniques.

To avoid averaging the partial volume effects resulting from bone structures with high Hounsfield unit (HU) values, skull stripping (brain extraction) was performed on each NCCT dataset prior to structure-preserving interpolation. The brain extraction is a six-step process, which was implemented using the Insight Segmentation and Registration Toolkit (ITK, https://itk.org/). The framework used for this follows the approach described by Muschelli *et al*.^20^, but was implemented in a slice-by-slice fashion. Therefore, each slice of the scan is first smoothed using a Gaussian filter (variance = 4 pixels), intensity thresholded between 0 and 100 HU, and then eroded using a circular structuring element (radius = 1 pixel). Subsequently, the largest connected component of the current slice is extracted and dilated with a circular structuring element (radius = 1 pixel). Once all slices of the scan are processed, any holes in the final mask are filled using ITK’s VotingBinaryHoleFillingImageFilter. The resulting brain masks were visually inspected for segmentation errors and manually modified as necessary.

### Atlas generation

The Advanced Normalization Tools (ANTs, http://stnava.github.io/ANTs/)^21^ were used for atlas generation. The ANTs package has tools for generating atlases for the same patient cohort with different imaging modalities (the antsMultivariateTemplateConstruction.sh script). This script was used for each modality independently to make use of the added control in warping the images. In this script, a random image is chosen to serve as the initial template to create a rigid-only atlas that is subsequently used to initialize the actual non-linear atlas formation. As a (default) pre-processing step, the script also performs a N4 bias correction^22^ prior to the actual atlas generation routine.

### Asymmetric atlas: parameter settings

Except the intensity normalisation, all other parameters required by the atlas generation script were identical for the FLAIR and NCCT atlas generation. Only for the NCCT atlases, the default template normalisation routine was turned off across all iterations to retain the true average HU values for each voxel.

To avoid translations of the atlas through the iterations, the atlas updates were performed using the affine deformation without the rigid component, followed by, the non-linear deformations for each iteration of the atlas generation process. The non-linear atlas formation was conducted using four iterations of the symmetric diffeomorphic registration with probability mapping as the similarity metric. In each registration iteration, 30 × 50 × 20 maximum cycles of deformations (coarse to fine) were applied.

Although these options are the default parameters, the other similarity metrics for intramodal registrations (such as cross-correlation, mean squared difference, and sum of squared differences) and other non-linear registration schemes were tested for improvement in atlas quality. By visual inspection, it was found that the default options of the ANTs script yielded the best results.

### Symmetric atlas: processing steps

Although the brain is inherently asymmetric, the scanning-induced distortion in the atlas may bias the findings of applied scanner-independent research, posing the need for symmetric atlases. This is accomplished by obtaining the average of the transformed flipped (x-axis) asymmetric atlas version and the asymmetric atlas in its native orientation. The symmetric atlas is devoid of tissue asymmetry across hemispheres and imaging-related asymmetry in the dataset.

### Post-processing

Different approaches for post-processing of the FLAIR and NCCT atlas were used. The FLAIR atlas was created using the individual FLAIR datasets without skull stripping because obtaining accurate and consistent brain extractions across all datasets was not possible. Instead, the Brain Extraction Toolkit (BET, FSL)^23^ was used for initial skull stripping of the generated FLAIR atlas by manually tuning the skull stripping parameters. The segmentation errors, mostly located in the posterior brain lobes, were corrected by converting the surface of the brain (including posterior patches of skull) into a surface mesh and manually smoothing the surface mesh in MeshLab (http://www.meshlab.net/)^24^. The manually corrected brain surface mask was converted back into a binary volumetric mask using ITK. The NCCT atlas was sharpened using the Laplacian filter to overcome the compounded averaging effect while retaining the HU values.

Lastly, in addition to the pre-processing routine, one final bias field correction was applied to both the FLAIR and NCCT atlases using the default parameters provided in the ANTs toolkit^22^. Subsequently, the FLAIR and NCCT templates were registered using the symmetric diffeomorphic method with mutual information as the similarity metric and Lanczos windowed sinc interpolator in the ANTs package.

## Data Records

The age-specific FLAIR and NCCT atlases are provided in niftii format at 1 mm³ isotropic resolution. These image files include the FLAIR MRI atlases, brain volumes, and respective brain masks (miplab-flair_asym_with-skull.nii.gz, miplab-flair_sym_with-skull.nii.gz; miplab-flair_asym_brain.nii.gz; miplab-flair_sym_brain.nii.gz; miplab-flair_asym_mask.nii.gz, miplab-flair_sym_mask.nii.gz respectively), the NCCT atlas (miplab-ncct_asym_brain.nii.gz, miplab-ncct_sym_brain.nii.gz), and the MNI deformations (miplab_to_mni_asym_warp.nii.gz, miplab_to_mni_sym_warp.nii.gz, mni_to_miplab_asym_warp.nii.gz, mni_to_miplab_sym_warp.nii.gz)^25^. The MNI deformations are composite transformations that include linear and non-linear deformations computed using the ANTs registration framework with the default parameters of the three stage (rigid-affine-deformable syn) registration. The FLAIR atlas is saved as float values, NCCT as integers, and masks as binary.

## Technical Validation

**Fig. 2:**
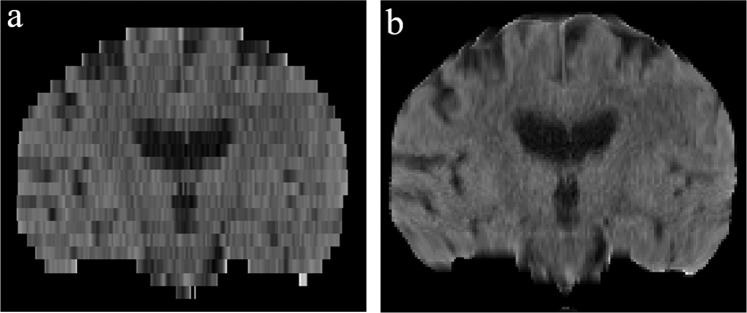
Effect of registration-based interpolation. (**a**) a sample dataset; (**b**) corresponding dataset after registration-based interpolation.

Figure 2 shows the impact of the applied registration-based interpolation method on the atlas generation process (keeping all other parameters constant). The reduction in the serration of hemispheric borders is evident in the registration-based sample dataset. The atlases created subsequently show a two-fold benefit. First, the boundaries of the brain at the apex are smooth, while the gyri and sulci edges are sharp and clearly visible. Second, and more importantly, the sulci around the insular lobe are more pronounced due to gray matter atrophy.

### Atlas evolution

The FLAIR and NCCT templates were created in a two-step process: initial atlas generation using rigid registrations followed by iterative non-linear shape updates. The initial atlas is a crude estimate of the (global) sample shape obtained by linearly aligning all scans in the dataset to the scan of a randomly selected subject. The resulting rigid group average serves as the initial template. Subsequently, in each iteration, all individual scans are diffeomorphically registered to this initial template. The average of the inverse of all these transforms is then applied to the initial atlas as a shape update for that iteration. The finer details of the sample shape and intensity of the template evolve over the four iterations. All intermediate deformations of each subject and the evolving group template were visually inspected after each iteration. The quality of the registration-based interpolation was ensured to be similar across all subjects prior to atlas generation.

**Fig. 3:**
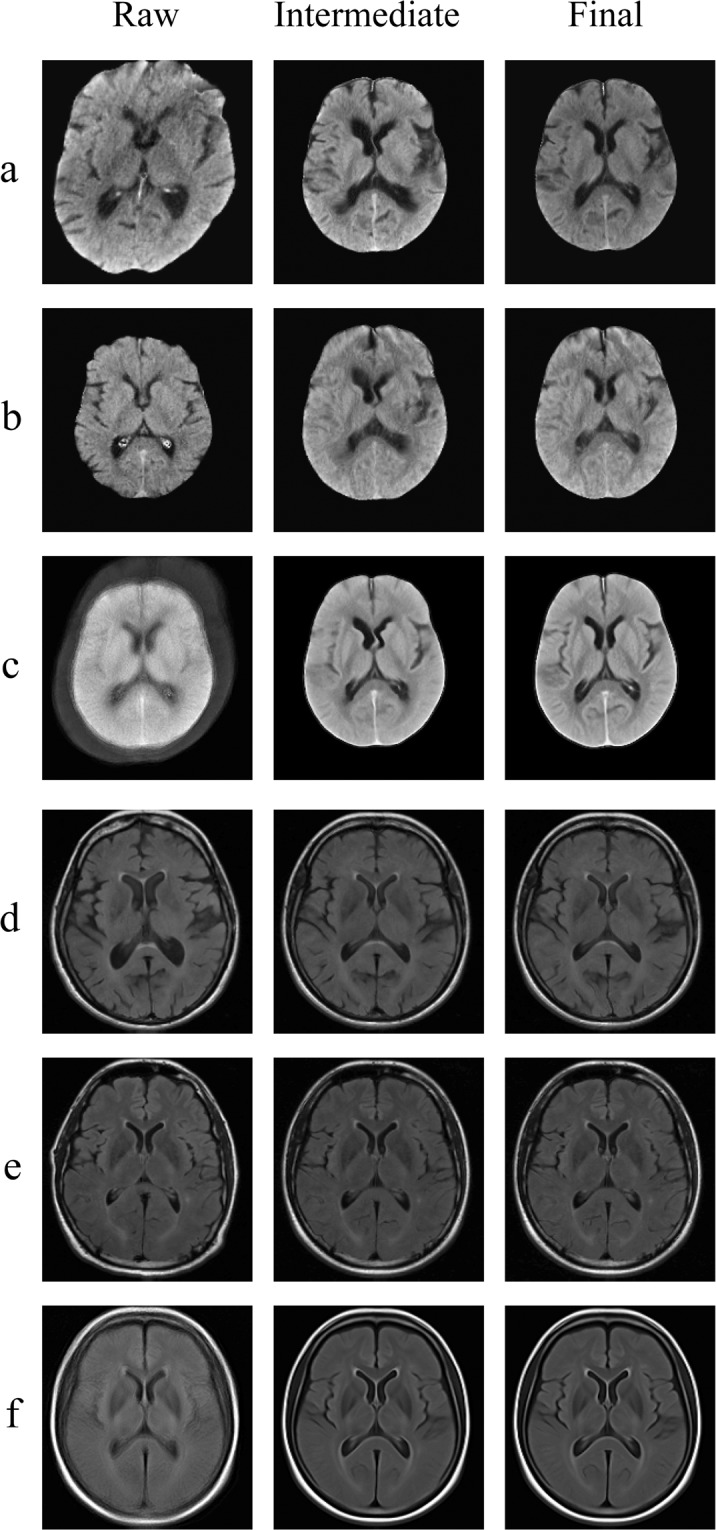
Atlas generation. (**a**) sample NCCT with large ventricles; (**b**) sample NCCT with small ventricles; (**c**) MIPLAB-NCCT atlas; (**d**) sample FLAIR with large ventricles; (**e**) sample FLAIR with small ventricles; (**f**) MIPLAB-FLAIR atlas.

Figure 3 shows this evolution of FLAIR and NCCT datasets for two subjects, one with large ventricles and extensive gray matter atrophy and a second with relatively small ventricles. The two datasets with large ventricles (Fig. 3a,d) are gradually transformed to the average ventricle size. Similarly, the datasets with small ventricles (Fig. 3b,e) are gradually transformed to the larger average ventricle size. The evolution of the non-linear shape updates for the FLAIR and NCCT templates are shown (Fig. 3c,f), respectively.

### Entropy

Image entropy is a quantitative metric to assess the sharpness of an image with visual assessment being its qualitative counterpart^26^. Briefly described, entropy provides a measure of randomness in the image. Thus, the images that lack sharp distinctions between brain regions would have high entropy values. Within the context of comparing representative group averages, the lower the entropy of an atlas, the more defined the sub-regions of the atlas are likely to be displayed and, therefore, should enable improved image correspondences after registration.

**Fig. 4:**
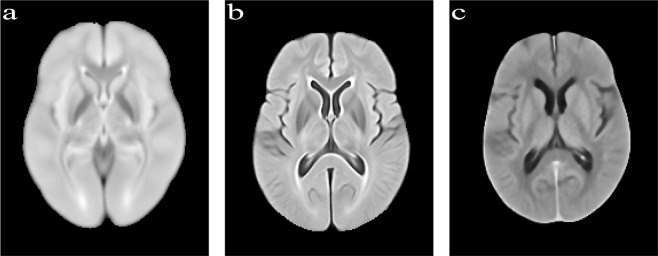
The MIPLAB atlases revealing relatively more detail in boundary regions. (**a**) Brainder-FLAIR atlas; (**b**) MIPLAB-FLAIR atlas; (**c**) MIPLAB-NCCT atlas.

To calculate the image entropy, each atlas was converted into a histogram of 256 bins. The histogram was subsequently normalised such that each bin represents the ratio of frequency of an intensity value to the sum of all frequencies of all 256 grayscale intensities. Entropy is the negative sum of the probabilities in each bin scaled by the natural log of each probability measure. Table 1 summarises the entropies of the proposed atlas in comparison to another available atlas often used in neuroimaging studies. The proposed MIPLAB-FLAIR atlas has a 60% reduction in entropy (*i.e*. better detail and sharpness of the image) in comparison to the Brainder FLAIR atlas. The proposed MIPLAB-NCCT atlas also has a low entropy value indicating that cerebral regions are sharp. Figure 4 shows slices displaying the basal ganglia structures from the atlases for visual inspection. In the MIPLAB-FLAIR atlas, the contrast distinction between the deep gray matter structures depicting the variation in iron content is evident. In the MIPLAB-NCCT atlas, the widening of the sulci around the insula as a result of cerebral atrophy is evident. Generally, both atlases show increased CSF attributed to healthy aging.

**Table 1:** Summary of the characteristics of the MIPLAB atlases in comparison with atlases previously published.

Atlas	Age (mean ± std)	Sample Size	Entropy
Brainder-FLAIR	48.4 ± 14.81	853	2.29
MIPLAB-FLAIR	65.9 ± 13.05	136	1.45
MIPLAB-NCCT	71.9 ± 14.04	47	1.55

## Usage Notes

The atlases provided are in slice correspondence with the standard MNI 152 nonlinear 2009c atlases. For each modality, the asymmetric and symmetric versions of the atlases are provided. Furthermore, the composite deformation fields that map the MNI atlases to the proposed MIPLAB atlases are also provided. The deformation fields can be applied to any atlas (or other information) that is defined in the MNI co-ordinate space to generate a corresponding age-matched version or to transfer segmentation data. All files are available in the Niftii data format and named as per the standard BIDS convention.

The Niftii data format allows using the atlas data in various tools used for neuroimage analysis such as Slicer, Free Surfer, or FSL. Multiple freely available tools can be used for registration such as ANTs and NiftyReg. These atlases can also be used in brain-mapping toolkits such as LESYMAP and MRICRON.

## Data Availability

The main template creation script used in this work is the antsMultivariateTemplateConstruction.sh script from ANTs. The auxiliary scripts for preprocessing and post processing are mostly implemented in ITK. The entire code-base used to build the FLAIR and NCCT atlases are provided in the GitHub repository (https://github.com/deepthirajashekar/FLAIR-and-NCCT-atlas-for-elderly). The pre-processing module contains the C++ code to perform the CT brain extraction and the registration-based interpolation. The atlas generation module is a bash script with the calls to the ANTs package and the specific parameters for FLAIR and NCCT. The post-processing module is a bash script that invokes various ANTs and FSL packages to generate the symmetric counterparts of the atlases and to obtain the deformation fields for the MNI atlases. The repository also includes the final templates (asymmetric FLAIR, symmetric FLAIR, asymmetric CT, symmetric CT), masks, and deformation fields to the MNI space. Additionally, the same information is made available on the official MIPLAB website (https://www.ucalgary.ca/miplab/downloads).
